# Clinicopathological prognostic factors for survival in patients with breast cancer: a retrospective study from a tertiary cancer centre from North-West India

**DOI:** 10.3332/ecancer.2025.1865

**Published:** 2025-03-06

**Authors:** Niketa Thakur, Ramandeep Singh, Sunigdha, Rajiv Devgan, Abhishek Sharma

**Affiliations:** 1 Department of Radiation Oncology, All India Institute of Medical Sciences, Bilaspur, HP 174037, India; 2 Department of Radiation Oncology, Government Medical College, Amritsar 143001, India; 3 Department of Anaesthesia and Critical Care, Sri Guru Ram Das Institute of Medical Sciences and Research, Amritsar 143501, India

**Keywords:** breast cancer, clinicopathological, prognostic factors, survival

## Abstract

**Background:**

Among women worldwide, breast cancer is the most prevalent malignancy. Understanding potential prognostic indicators is critical for selecting the optimal treatment modalities, evaluating the response and creating a follow-up plan. In this retrospective study, the clinical and pathological features of primary breast cancer patients who presented to the tertiary cancer centre in the past 10 years were retrospectively studied and analysed to assess their correlation with survival.

**Patients and methods:**

Histopathologically confirmed breast cancer patients presenting between January 2014 and December 2023 were included in this study. Clinical data and treatment details including the surgical procedure, chemotherapy and radiation treatment were collected from the medical records. The study utilised both univariate and multivariate Cox proportional hazards models to determine the most significant independent prognostic variables for overall survival (OS).

**Results:**

A total of 676 patients were treated for breast cancer in our centre between January 2014 and December 2023. The median age was 50 years. Three seventy nine patients (56.0%) presented with early breast cancer at the time of diagnosis while 272 patients (40.2%) had locally advanced breast cancer corresponding to clinical stage III. Twenty one (3.1%) patients presented with stage IV. In the univariate analysis, factors significantly associated with OS were stage at presentation, grade, positive lymph node status, number of positive lymph nodes, presence of lymphovascular space invasion (LVSI), presence of perinodal extension (PNE) and distant metastasis at presentation. On multivariate analysis, grade of tumor, LVSI and PNE were significant factors affecting survival outcome.

**Conclusion:**

Clinical stage at presentation, tumour grade, lymph node status, presence of LVSI, PNE and tumour grade were the prognostic variables substantially associated with survival. A comprehensive approach that includes early detection and appropriate treatment modalities tailored to individual patient characteristics is essential for optimising survival outcomes in breast cancer patients.

## Introduction

Among women worldwide, breast cancer is the most prevalent malignancy. It has now surpassed lung cancer as the leading cause of global cancer in 2020, with an estimated 2.3 million new cases, representing 11.7% of all cancer cases followed by lung (11.4%), colorectal (10.0%), prostate (7.3%) and stomach (5.6%) cancers [1]. Among females, it is the most common cause of cancer death, followed by lung and colorectal cancer.

Breast cancer is a complex disease influenced by multiple factors that contribute to its development. The disease is prevalent globally, although its incidence, death rates and survival rates varied significantly across various regions, possibly due to multiple reasons, which could be due to many factors such as population structure, lifestyle, genetic factors and environment [2, 3]. With the advent of population growth, changes in lifestyle and migration from rural to urban areas, there is an increase in the incidence of breast carcinoma in developing countries. The nonmodifiable risk factors include genetic mutation, age and family history of breast carcinoma, while the other risk factors include reproductive factors such as early age at menarche and late age at menopause, nulliparity, older age at first full-term birth, number of children and duration of breastfeeding [4, 5]. Hereditary causes account for only 5%–10% of breast cancer cases, while germline mutations in breast cancer gene (BRCA)1 or BRCA2 account for 30% of inheritable breast cancer cases [6].

Understanding potential prognostic indicators is critical for selecting the optimal treatment modalities, evaluating the response and creating a follow-up plan [7]. In this study, the clinical and pathological features of primary breast cancer patients who presented to the tertiary cancer centre in the past 10 years were retrospectively studied and analysed to assess their correlation with survival.

## Patients and methods

Histopathologically confirmed breast cancer patients presenting between January 2014 and December 2023 were included in this study. Each patient underwent baseline blood investigations followed by mammography or sono‑mammography. The diagnosis of breast cancer was established by using core biopsy. Clinical data and treatment details including the surgical procedure, chemotherapy and radiation treatment were collected from the medical records. Relevant data, such as tumour type, size, grade, nodal status, presence of lymphovascular space invasion (LVSI) and perinodal extension (PNE), were noted and evaluated from the histopathology reports. For each patient, the status of the HER-2 Neu receptor, progesterone receptor and oestrogen receptor was determined. **The impact of various clinicopathological and treatment-related factors including age, clinical stage, tumor grade, tumor size, histological subtype, number of positive lymph nodes, extracapsular extension,** LVSI**, distant metstasis at diagnosis, type of surgery, chemotherapy received or not, radiotherapy received or not, hormone receptor status, Her 2 neu status, on overall survival (**OS) **was analysed in this study.** Follow‑up information was collected from hospital records. **Patients with incomplete data or those lost to follow-up were excluded from the study.** The study protocol was approved by the Institutional Ethics Committee.

**In patients treated with curative intent, chemotherapy schedule followed was four cycles of doxorubin/epirubicin and cyclophosphamide followed by four cycles of taxane-based chemotherapy. After response from the neoadjuvant chemotherapy, the patients underwent surgery either breast conservation surgery (BCS) or modified radical mastectomy (MRM) depending on the indication. The subsequent cycles were given as adjuvant chemotherapy. After completion of chemotherapy patients were considered for radiotherapy depending on the indication. All post MRM patients in whom radiotherapy was indicated received radiation therapy to the chest wall with or without the inclusion of ipsilateral supraclavicular fossa. The dose prescribed was 5,000 cGy in 25 fractions over a period of 5 weeks or 4,000 cGy in 15 fractions over 3 weeks. All patients who underwent BCS received radiotherapy to conserved breast with or without the inclusion of supraclavicular fossa to a dose of 4,000 cGy in 15 fractions over 3 weeks followed by tumor bed boost to a dose of 1,000 cGy in 5 fractions.**

Statistical analysis was performed with SPSS statistical software version 25. The baseline clinical characteristics were presented in absolute and percentage figures and were calculated using descriptive statistics. Log-rank tests were used to assess the statistical significance of the discrepancies between the survival curves. **OS was defined as the period from the date of diagnosis to death or last follow-up**. The study utilised both univariate and multivariate Cox proportional hazards models to determine the most significant independent prognostic variables for OS. A *p* value of <0.05 was considered as statistically significant.

## Results

**A total of 701 breast cancer patients were treated at our center between January 2014 and December 2023. Of these, 25 patients were excluded due to incomplete data, resulting in a final cohort of 676 evaluable patients.** The median age was 50 years. Among 676 patients with breast cancer, the proportion of patients greater than 40 years of age was 561 (83%) with the majority of them in the age group of 41–50 years of age (36.5%) (Table 1).

### Clinical features of the patients

The baseline clinical characteristics are mentioned in Table 1. The majority of the patients presented with a symptomatic lump (97%) with most of them having left-sided tumors (53.7%). One patient had synchronous bilateral breast cancer corresponding to the incidence of 0.1**%. Twelve patients had a family history of breast cancer, corresponding to an incidence of 1.8%.** No patient underwent BRCA analysis. Three seventy nine patients (56.0%) presented with early breast cancer (clinical stage I and II) at the time of diagnosis. Nearly, 272 patients (40.2%) had locally advanced breast cancer corresponding to clinical stage III. Twenty one (3.1%) patients presented with stage IV at diagnosis with bone as the most common site of distant metastatis (1.4%). **Luminal A was the most common molecular subtype accounting for 37% of the patients.**

### Histopathological details

The final histology revealed a median tumour size of 3 cm. Six forty two patients (95%) had infiltrating ductal carcinoma while the remaining had other histologies (Table 1). A total of 439 patients had positive lymph nodes (64.9%) of which 200 patients (45.5%) had more than 3 lymph nodes positive. Out of the total number of patients, 48 (7.1%) had grade I tumours, 310 (45.8%) had grade II tumours and 318 (47%) had grade III tumours. Two sixty-four patients (39%) had LVSI, while 176 patients (26%) had PNE. Out of the total number of patients, 337 individuals, accounting for 49.8% of the sample, tested positive for hormone receptors. Her-2 Neu receptors were found to be positive in 20 patients, accounting for 11.39% of the total. Additionally, 186 individuals were identified as having triple negative receptors, representing 27.5% of the total.

### Treatment details

Of the 662 patients who underwent surgery, 588 (88.8%) patients underwent MRM and 74 (11.1%) patients underwent BCS. A total of 621 (91.8%) patients received chemotherapy. Of the 621 patients, 20 (3.2%) patients received chemotherapy with palliative intent due to their disease status. Adjuvant loco-regional radiation was given to 628 patients, which accounted for 92.8% of the total patients. **Of the 337 patients who were hormone receptor positive, 320 (95%) patients received hormone therapy. Among 20 patients who were Her 2 neu positive, 15 (75%) patients received trastuzumab.**

### Prognostic factors for survival

The median follow‑up period for all the patients was 56 months. In the univariate analysis (Table 2), factors significantly associated with OS were stage at presentation (HR = 0.712; 95% CI = 0.546–0.928), grade (HR = 3.708; 95% CI = 2.739–5.021), positive lymph node status (HR = 0.727; 95% CI = 0.542–1.378), number of positive lymph nodes (HR = 0.712; 95% CI = 0.546–0.928), presence of LVSI (HR = 0.712; 95% CI = 0.546-0.928), presence of PNE (HR = 0.197; 95% CI = 0.149–0.259), distant metastasis at presentation (HR = 0.268; 95% CI = 0.156–0.461), **treatment-related factors such as chemotherapy (HR = 4.593; 95% CI = 3.230–6.532) and radiotherapy (HR = 8.543; 95% CI = 6.051–12.062).** The age at diagnosis, tumor size, hormone and Her 2 neu receptor status did not have any significant impact on survival.

**Figure 1.**
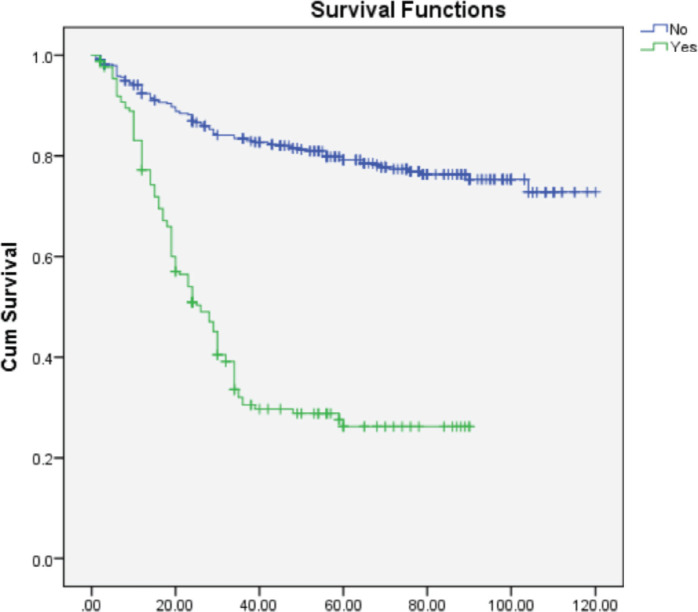
Impact of PNE on OS.

**Figure 2.**
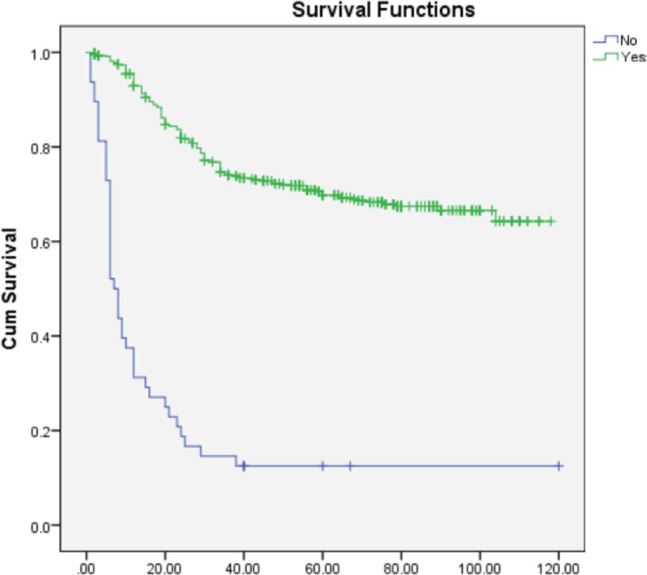
Comparison of OS in patients receiving chemotherapy versus not receiving chemotherapy.

**Figure 3.**
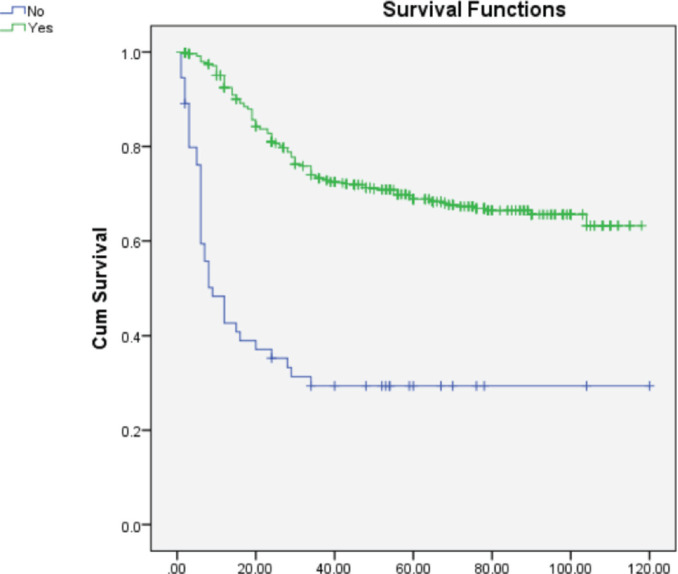
Comparison of OS in patients receiving radiotherapy versus not receiving radiotherapy.

On multivariate analysis (Table 2), grade of tumor (HR = 2.268; 95% CI = 1.892–3.649), LVSI (HR = 0.570; 95% CI = 0.366–0.889), PNE (HR = 0.308; 95% CI = 0.196–0.484) **(**Figure 1**)** and **treatment-related factors such as chemotherapy (HR = 3.265; 95% CI = 2.193–4.861) (**Figure 2**) and radiotherapy (HR = 6.428; 95% CI = 3.970–9.970) (**Figure 3**)** were significant factors affecting survival outcome.

## Discussion

Breast cancer is the most common cancer among women in both developed and developing countries [1, 8, 9]. Reports from the western world show that female breast carcinoma is predominantly seen in the fifth and sixth decade [10–12]. In India, carcinoma breast incidence peaks among women at a younger age as compared to women from Western countries [13, 14]. Some research studies conducted earlier in India have observed age at diagnosis of breast cancer between 45 and 50 years [15, 16]. We also found almost similar results in our study in which the majority of patients are in the age group of 41–50 years (36.5%). Currently, the incidence of breast cancer among women under the age of 40 accounts for roughly 5%–7.5% of the total annual diagnoses in Western Europe and the United States [17]. The occurrence is twice as high in Asian studies [13]. In our study, the proportion of breast cancer patients below 40 years of age was 17.0%. The incidence of breast carcinoma in males was found to be 1.0%, similar to other reports published in the literature [18, 19]. In a study by Nair *et al* [16], the male breast carcinoma incidence was 0.95% [16]. The most common side of breast cancer was left accounting for 53.7% of the cases. Thakur *et al* [20] in their series on dual malignancy, reported the breast as the most common site of first and second primary with patients of synchronous bilateral breast cancer constituting 13.0% of the total cases with dual malignancy. However, in our sample, only one patient had synchronous bilateral breast cancer.

In a study by Nair *et al* [16], 40.5% of patients presented with early breast cancer. In Sofi *et al* [21], 3% of the total patients presented with metastatic. In our study, 56% of the patients were of early stage while 3.1% of patients presented with distant metastasis at diagnosis. Previous research studies on stage of breast cancer have reported that more than 50% of newly diagnosed patients presented with stage III or IV breast cancer [22, 23]. In the present study, 43.3% of patients presented with stage III or IV.

In a review of 3,602 women who underwent surgery for early breast cancer, de Bock *et al* [24] demonstrated the impact of young age on local relapse. The results of multivariate analysis showed that younger age and breast conservation were risk factors for isolated loco-regional recurrence. Multiple studies have demonstrated that the prognosis of breast cancer in younger women is often poorer compared to older women. This is primarily due to the fact that younger women tend to be diagnosed at a late stage and have more aggressive tumour features [25–27]. In our study also, there was a trend towards decreased survival in patients younger than 40 years of age.

Invasive ductal carcinoma was the most common histology found in our study accounting for 97% of the patients with Luminal A as the most common molecular type with 37% of the patients. This study is consistent with the large-sample data previously reported for age at diagnosis, tumor type, stage and molecular classification [14, 16, 22, 28, 29]. Nguyen *et al* [30] reported on 793 patients with invasive breast cancer who received breast-conserving therapy and radiation. With a median follow-up of 70 months, the 5-year rate of local recurrence was 0.8% for luminal A, 1.5% for luminal B, 8.4% for HER2 and 7.1% for basal. In addition, on multivariate analysis, HER2 and basal subtypes were associated with increased local recurrence as compared with luminal A (*p* < 0.01). In our study also, higher local recurrence rates were seen in Her 2 enriched and basal subtypes.

Several studies have indicated that patients with hormonal receptors have a significantly higher survival rate. Crowe *et al* [31] studied 1,392 patients with carcinoma of the breast treated with MRM. ER positive tumors (≥3 fmol/mg cytosol protein) were found in 1,063 patients (76.4%). Their 10-year OS rate of 65.9% was significantly better than the 56% rate in 329 patients with ER-negative tumors (*p* = 0.0001). Similarly, our study also reported a trend toward higher survival in ER positive tumors than ER negative tumors.

Overexpression of the Her 2 neu protein is associated with tumor aggressiveness and decreased disease-free survival in node-positive patients, with variable prognostic significance among node-negative patients. Miles *et al* [32] examined the relationship between HER2/neu status and outcome in 274 node-positive women who were randomised to receive six cycles of adjuvant CMF (cyclophosphamide, methotrexate, 5FU) or no adjuvant therapy. Although all of the treated women appeared to benefit from adjuvant CMF, the improvement in survival was less in the HER2/neu-positive patients. In our study, Her 2 neu positivity was associated with a trend towards poor OS.

The prevalence of lymph node involvement exhibits significant variation across different studies, ranging from 40% to 61% [33, 34]. The results of our study indicated that 56.9% of patients had positive lymph nodes. Studies have shown higher rates of local recurrence in those with three or more lymph nodes compared with N0 or N1a lymph nodes [35]. This is consistent with the findings of our study in which patients having more than 3 lymph nodes had significantly poor survival outcomes.

The LVSI has also been associated with a bad prognosis. Rosen *et al* [36] observed a correlation between LVSI and the risk of recurrence and death after 20 years of follow-up. The LVSI positivity rate in our study was 39%, and this had a significant impact on survival. Similarly, PNE also significantly affected survival outcomes.

**Although tumor size and receptor status are well-documented prognostic factors in breast cancer, they did not demonstrate a statistically significant impact on survival in our cohort. The patient cohort had a relatively homogenous distribution of tumor sizes, leading to a lack of sufficient variability to detect a significant impact on survival. Moreover, a substantial proportion of patients had received effective systemic, hormonal and target therapy that mitigated the influence of tumor size and receptor status on survival outcomes.**

**The results of our study can play a significant role in guiding adjuvant treatment. These findings can aid clinicians in stratifying patients into distinct risk categories, which is essential for tailoring treatment strategies. For instance, patients with advanced clinical stages or high-grade tumors may benefit from more aggressive systemic therapies and close monitoring. The presence of lymphovascular invasion and extracapsular extension could indicate a higher risk of recurrence, prompting consideration of adjuvant therapies such as radiotherapy or chemotherapy. The significance of extracapsular extension in multivariate analysis underscores the importance of thorough surgical evaluation of lymph nodes as it may warrant intensified adjuvant therapy such as adjuvant radiotherapy including regional nodal irradiation and chemotherapy to improve survival outcomes. High-grade tumors and lymphovascular invasion emphasize the need for adjuvant systemic therapies to address potential micrometastatic disease. Patients with these adverse prognostic features may benefit from more frequent follow-up visits and imaging to detect early signs of recurrence. The strong association between clinical stage and survival highlights the need for public health initiatives aimed at early detection. Educating the community and improving access to screening programs could reduce the proportion of patients presenting with advanced disease, ultimately improving survival rates.**

There are some limitations of this study. The study was conducted at a single center which may limit the generalisability of the findings to other populations or healthcare settings. The study had a retrospective design, relying on data collected from patient records. The study includes patients treated for breast cancer within a specific timeframe. The median follow-up period may be relatively short for assessing long-term survival outcomes. While the study describes treatment modalities administered to patients, there may be variability in treatment protocols and adherence across different clinicians or time periods. Variations in treatment approaches could confound the analysis of treatment effects on survival outcomes. **Also, no BRCA analysis was performed on any patients despite family history cases.** This might be due to the fact that **at the time of data collection, BRCA testing was not routinely available at our center and also due to limited accessibility to genetic testing in the region. BRCA analysis has gained wider clinical acceptance and availability in recent years, and we agree that its inclusion could have added significant value to understanding hereditary breast cancer risk in this cohort. However, our study focused on clinicopathological prognostic factors that were feasible to assess retrospectively within the given resource constraints. Future prospective studies may incorporate BRCA testing to provide a more comprehensive evaluation of familial and genetic influences on breast cancer outcomes. Besides this, there is the absence of quality-of-life (QoL) assessments in our study. As this was a retrospective analysis, patient-reported outcomes, including QoL data, were not collected at the time of treatment. While our study primarily focused on clinicopathological prognostic factors and survival outcomes, QoL assessments would have provided valuable insights into the impact of treatments on patients’ overall well-being.** Hence, large multicentric trials are required to provide more robust evidence for informing clinical practice and further research in breast cancer management.

## Conclusion

Clinical stage at presentation, tumour grade, lymph node status, presence of LVSI, PNE and tumour grade were the prognostic variables substantially associated with survival. A comprehensive approach that includes early detection and appropriate treatment modalities tailored to individual patient characteristics is essential for optimising survival outcomes in breast cancer patients. Prospective validation studies are warranted to further elucidate the implications of these findings.

## Conflicts of interests

There are no conflicts of interests.

## Funding

No financial support was received from any organisation for the submitted work.

## Ethics committee

The research is hereby approved by the Institutional Ethics Committee.

Government Medical College, Amritsar Institutional Ethics Committee issued approval.

## Author contributions

**Table:** 

	Author 1	Author 2	Author 3	Author 4	Author 5
Concepts	✓				
Design	✓				
Definition of intellectual event	✓	✓	✓		
Literature search	✓	✓			
Clinical studies	✓	✓			
Experimental studies	✓	✓	✓	✓	
Data acquisition	✓	✓	✓	✓	
Data analysis	✓	✓	✓	✓	✓
Statistical analysis	✓	✓			✓
Manuscript preparation	✓				
Manuscript editing	✓	✓	✓		✓
Manuscript review	✓	✓		✓	✓
Gaurantor	✓				

## Figures and Tables

**Table 1.** Patient characteristics.

Clinical or pathological feature	*n* (%)
Gender	
Female	669 (98.9%)
Male	7 (1.0%)
Age	
<40	115 (17%)
≥40	561 (83%)
Laterality	
Right	312 (46.2%)
Left	363 (53.7%)
Bilateral	1 (0.1%)
Marital status	
Married	672 (99.4%)
Unmarried	4 (0.5%)
Menopausal status	
Premenopausal	180 (23.6%)
Postmenopausal	496 (73.4%)
Parity	
Nulliparous	12 (1.8%)
Parous	664 (98.2%)
Family history (first and second degree relatives)	
Yes	12 (1.8%)
No	664 (98.2%)
Stage	
Stage 1	40 (5.9%)
Stage 2	339 (50.1%)
Stage 3	272 (40.2%)
Stage 4	21 (3.1%)
Molecular subtype	
Luminal A	250 (37%)
Luminal B	95 (14.1%)
Her 2 enriched	145 (21.4%)
Triple negative	186 (27.5%)
Histology	
Invasive ductal carcinoma	642 (95%)
Phylloides	5 (0.7%)
Neuroendocrine	2 (0.2%)
Lobular carcinoma	8 (1.18%)
Metaplastic carcinoma	2 (0.2%)
Papillary carcinoma	6 (0.8%)
DCIS	8 (1.2%)
Medullary	3 (0.4%)
Grade	
I	48 (7.1%)
II	310 (45.8%)
III	318 (47%)
Distant metastasis at presentation	
Brain	8 (1.2%)
Bone	10 (1.4%)
Visceral	3 (0.4%)

**Table 2.** Univariate and multivariate analysis of prognostic factors affecting survival.

	Univariate analysis			Multivariate analysis		
Variables (*n*)	HR	*p* value	95% CI	HR	*p* value	95% CI
Age in years ≤40 >40	0.816	0.236	0.583–1.143	0.788	0.194	0.549–1.129
Clinical stage Stage I & II Stage III & IV	0.712	0.012	0.546–0.928	0.948	0.787	0.643–1.396
Grade of the primary tumor I & II III	3.708	<0.001	2.739–5.021	2.628	<0.001	1.892–3.649
Tumor size ≤2 >2	0.908	0.651	0.599–1.378	0.996	0.986	0.634–1.564
Lymph node status Positive Negative	0.727	0.034	0.542–0.976	0.792	0.199	0.554–1.131
Number of lymph nodes positive ≤3 >3	1.438	0.010	1.090–1.898	1.093	0.626	0.765–1.561
PNE Yes No	0.197	<0.001	0.149–0.259	0.308	<0.001	0.196–0.484
Lymphovasular space invasion Yes No	0.125	<0.001	0.163–0.283	0.570	0.013	0.366–0.889
Distant metastasis at diagnosis Yes No	0.268	<0.001	0.156–0.461	1.133	0.739	0.544–2.362
Hormone receptor Positive Negative	0.851	0.235	0.651–1.111	0.912	0.513	0.691–1.203
Her 2 neu Positive Negative	1.300	0.062	0.987–1.713	1.261	0.118	0.943–1.686
Chemotherapy Received Not received	4.593	<0.001	3.230–6.532	3.265	<0.001	2.176–4.921
Radiotherapy Received Not received	8.543	<0.001	6.051–12.062	6.428	<0.001	3.970–9.970

## References

[1] Bray F, Ferley J, Soeriomataram I (2018). Global cancer statistics 2018: GLOBOCAN. Estimates of incidence and mortality worldwide for 36 cancers in 185 countries. CA Cancer J Clin.

[2] Zendehdel M, Niakan B, Keshtkar A (2018). Subtypes of benign breast disease as a risk factor for breast cancer: a systematic review and meta-analysis protocol. Iran J Med Sci.

[3] Hortobagyi GN, Garza Salazar J, Pritchard K (2005). The global breast cancer burden: variations in epidemiology and survival. Clin Breast Cancer.

[4] Rose DP, Vona-Davis L (2010). Interaction between menopausal status and obesity in affecting breast cancer risk. Maturitas.

[5] Hemminki K, Försti A, Sundquist J (2011). Preventable breast cancer is postmenopausal. Breast Cancer Res Treat.

[6] Valencia OM, Samuel SE, Viscusi RK (2017). The role of genetic testing in patients with breast cancer: a review. JAMA Surg.

[7] Thakur N, Ghoshal S (2019). Impact of early palliative care on survival in advanced-stage cancer patients: an institution based retrospective cross-sectional study. Asian Pac J Cancer Care.

[8] Siegel RL, Miller KD, Jemal A (2016). Cancer statistics. CA Cancer J Clin.

[9] Miller KD, Siegel RL, Lin CC (2016). Cancer treatment and survivorship statistics. CA Cancer J Clin.

[10] Li Y, Li Q, Mo H (2021). Incidence, risk factors and survival of patients with brain metastases at initial metastatic breast cancer diagnosis in China. Breast.

[11] Le Guennec D, Rougé S, Caldefie-Chézet F (2020). Obesity and breast cancer: two diseases of aging limited by physical activity. Med Sci (Paris).

[12] Price TR, Friedenreich CM, Robson PJ (2020). High-sensitivity C-reactive protein, hemoglobin A1c and breast cancer risk: a nested case-control study from Alberta's Tomorrow Project cohort. Cancer Causes Control.

[13] Agarwal G, Pradeep PV, Aggarwal V (2007). Spectrum of breast cancer in Asian women. World J Surg.

[14] Deshmukh SP, Mane AD, Zade BP (2014). Retrospective analysis of clinicopathological factors and outcome in breast cancer in young women in a tertiary care hospital in India. Indian J Cancer.

[15] Saxena S, Rekhi B, Bansal A (2005). Clinico-morphological patterns of breast cancer including family history in a New Delhi hospital, India – a cross-sectional study. World J Surg Oncol.

[16] Nair N, Shet T, Parmar V (2018). Breast cancer in India-an audit, with outcome analysis. Indian J Cancer.

[17] van der Hage JA, Mieog JS, Velde CJ (2011). Impact of established prognostic factors and molecular subtype in very young breast cancer patients: pooled analysis of four EORTC randomized controlled trials. Breast Cancer Res.

[18] Coughlin SS, Thompson TD, Hall HI (2002). Breast and cervical carcinoma screening practices among women in rural and nonrural areas of the United States, 1998–1999. Cancer.

[19] Joseph A, Mokbel K (2004). Male breast cancer. Int J Fertil Womens Med.

[20] Thakur N, Kaur H, Sudan M (2023). Clinicopathological analysis of patients with dual malignancies: a retrospective study. J Can Res Ther.

[21] Sofi NY, Jain M, Kapil U (2019). Epidemiological characteristics of breast cancer patients attending a tertiary health-care institute in the National Capital Territory of India. J Can Res Ther.

[22] Akhtar M, Akulwar V, Gandhi D (2011). Is locally advanced breast cancer a neglected disease?. Indian J Cancer.

[23] Chopra R (2001). The Indian scene. J Clin Oncol.

[24] de Bock GH, Hage JA, Putter H (2006). Isolated loco-regional recurrence of breast cancer is more common in young patients and following breast conserving therapy: long-term results of European Organisation for Research and Treatment of Cancer studies. Eur J Cancer.

[25] Winchester DP, Osteen RT, Menck HR (1996). The National Cancer Data Base report on breast carcinoma characteristics and outcome in relation to age. Cancer.

[26] Kollias J, Elston CW, Ellis IO (1997). Early‑onset breast cancer – histopathological and prognostic considerations. Br J Cancer.

[27] Walker RA, Lees E, Webb MB (1996). Breast carcinomas occurring in young women (<35 years) are different. Br J Cancer.

[28] Anderson WF, Chatterjee N, Ershler WB (2002). Estrogen receptor breast cancer phenotypes in the surveillance, epidemiology, and end results database. Breast Cancer Res Treat.

[29] El-Tamer MB, Wait RB (1999). Age at presentation of African-American and Caucasian breast cancer patients. J Am Coll Surg.

[30] Nguyen PL, Taghian AG, Katz MS (2008). Breast cancer subtype approximated by estrogen receptor, progesterone receptor, and HER-2 is associated with local and distant recurrence after breast-conserving therapy. J Clin Oncol.

[31] Crowe JP, Gordon NH, Hubay CA (1991). Estrogen receptor determination and long term survival of patients with carcinoma of the breast. Surg Gynecol Obstet.

[32] Miles DW, Harris WH, Gillett CE (1999). Effect of c-erb2 and estrogen receptor status on survival of women with primary breast cancer treated with adjuvant cyclophosphamide/methotrexate/fluorouracil. Int J Cancer.

[33] Fernandopulle SM, Cher‑Siangang P, Tan PH (2006). Breast carcinoma in women 35 years and younger: a pathological study. Pathology.

[34] McAree B, O’Donnell ME, Spence A (2010). Breast cancer in women under 40 years of age: a series of 57 cases from Northern Ireland. Breast.

[35] van Limbergen E, Bogaert W, Schueren E (1987). Tumor excision and radiotherapy as primary treatment of breast cancer. Analysis of patient and treatment parameters and local control. Radiother Oncol.

[36] Rosen PR, Groshen S, Saigo PE (1989). A long-term follow-up study of survival in stage I (T1N0M0) and stage II (T1N1M0) breast carcinoma. J Clin Oncol.

